# From trauma to depression: structural, synaptic, epigenetic, and molecular pathways linking early stress to lifelong vulnerability

**DOI:** 10.3389/fpsyt.2025.1666599

**Published:** 2025-10-16

**Authors:** Mina Takahashi, Richard C. Shelton

**Affiliations:** ^1^ Department of Psychiatry, Heersink School of Medicine, University of Alabama at Birmingham, Huntsville, AL, United States; ^2^ Department of Psychiatry and Behavioral Neurobiology, Heersink School of Medicine, University of Alabama at Birmingham, Birmingham, AL, United States

**Keywords:** abuse, adverse childhood experiences, a-Amino-3-hydroxy-5-methyl-4-isoxazolepropionic acid (AMPA) receptor, genomics, glutamate, hypothalamic-pituitary adrenal axis, micro RNAs

## Abstract

Major depressive disorder (MDD) is a complex and debilitating condition with high global prevalence. While pharmacological treatments are available, the long-term biological underpinnings – especially those linked to adverse childhood experiences (ACEs), remain incompletely understood. ACEs, including physical, sexual, and emotional abuse, neglect, and other traumas, significantly increase lifelong vulnerability to depression and reduce responsive to treatment. Epigenetic mechanisms such as DNA methylation and altered expression of mRNA and short and long non-coding RNAs such as microRNAs (miRNAs) are emerging as key mediators of the relationship between early environmental adversity and brain development and function. Specific miRNAs (e.g., *miR-124*, *miR-135*) influence neuroinflammation, and affect synaptic plasticity and monoaminergic signaling. Concurrently, DNA methylation in promoter regions can silence genes critical for stress regulation. For example, hypermethylation of the *NR3C1* gene (encoding the glucocorticoid receptor) has been linked with altered HPA axis feedback and cortisol imbalance following ACEs. These epigenetic changes, together with trauma-induced microglial activation and neuroinflammation, may create lasting neural vulnerability. This paper explores how the interplay between childhood trauma, hormonal dysregulation, microglial activation, and epigenetic modification contributes to the pathophysiology of depression. Synthesizing evidence across epigenetic networks and neurobiological systems can deepen an understanding of trauma-related mood disorders. This may inform targeted interventions, identify biomarkers for diagnosis and treatment, and support personalized approaches to care and suicide prevention.

## Introduction

Adverse childhood experiences (ACEs) such as abuse, neglect, loss, and other traumas, represent significant risk factors for subsequent mental illness and suicide. There is now strong evidence that childhood adversity, especially those that are experienced very early in life, induce longstanding physiological changes that underlie this risk. The purpose of this article is to review known physiological adaptations to adversity that are associated with these negative outcomes.

## Methods

This article is intended as an narrative review, providing and overview and summary of existing relevant literature. We searched the MEDLINE database (PubMed) for articles published before the original submission date of July 7, 2025. Searches included the following terms: adverse childhood experiences, childhood trauma, abuse (including physical, sexual and emotional), neglect AND the following: adrenocorticotrophic hormone (ACTH); α-amino-3-hydroxy-5-methyl-4-isoxazolepropionic acid (AMPA) receptor; amygdala; apoptosis; corticotropin releasing hormone (CRH), CRH receptor 1 (CRHR1); cortisol (and corticosterone); dendritic spine; depression/major depression (MDD); DNA methylation; epidemiology; epigenetics; excitotoxicity; FK506 binding protein 5 (FKBP5); functional magnetic resonance imaging (fMRI); glucorticoid; glutamate; glycogen synthase kinse 3 (GSK3); hippocampus; hypothalamic-pituitary-adrenal (HPAA) axis; hypothalamus; hypothalamus; inflammation (neuroinflammation); interleukin; interneuron; ketamine; locus coeruleus; micro RNA (miRNA); microglia; n-methyl-D-aspartic acid (NMDA) receptor; neural circuit; neuroplasticity; neurotrophin (including brain derived neurotrophic factor [BDNF]); post-traumatic stress disorder (PTSD); postmortem brain study; pruning; pyramidal cell; synapse; triggering receptor expressed on myeloid cells 2 (TREM2); tropomyosin-related kinase B (TrkB) receptors; and ventromedial prefrontal cortex (vmPFC). We also examined reference lists of relevant review papers, noted in the text. Appropriate papers were then selected by the authors for inclusion.

## Adverse childhood experiences: long-term risk factors for depression

ACEs refer to a range of serious challenges a child may face during critical developmental periods, including neglect; physical, sexual, or emotional abuse; poverty; discrimination; exposure to violence; and various forms of household dysfunction, such as living with family members who have substance use or mental health disorders, incarceration, or exposure to intimate partner violence (1–3). These adverse experiences can significantly shape brain and behavioral development, with consequences that extend well into adulthood (4, 5). Approximately one in five adolescents in the United States have experienced ACEs at some point during childhood (6, 7). The Centers for Disease Control and Prevention (CDC) defines ACEs as potentially traumatic events occurring before the age of 18 (8). Approximately two-thirds of U.S. adults report having experienced at least one ACE, and one in six reports experiencing four or more. Adults who experienced four or more ACEs were 4.6 times more likely to report depression in the past year, compared to those with no ACEs and even experiencing just one ACE increased the risk of depression by 50% (8). Felitti et al. (9) identified a dose-response relationship: the more adverse experiences a person had in childhood, the more likely they were to suffer from serious health issues in adulthood, including cardiovascular disease (CVD) (10–13), diabetes (14), high cholesterol, a known contributor to CVD (15), cognitive and other neurological disorders (16), substance use disorders, and internalizing problems such as anxiety and depression (17, 18). Evidence also shows that preventing or addressing ACEs early could reduce the overall prevalence of depression in the population by up to 44.1% (18).

**Figure 1:**
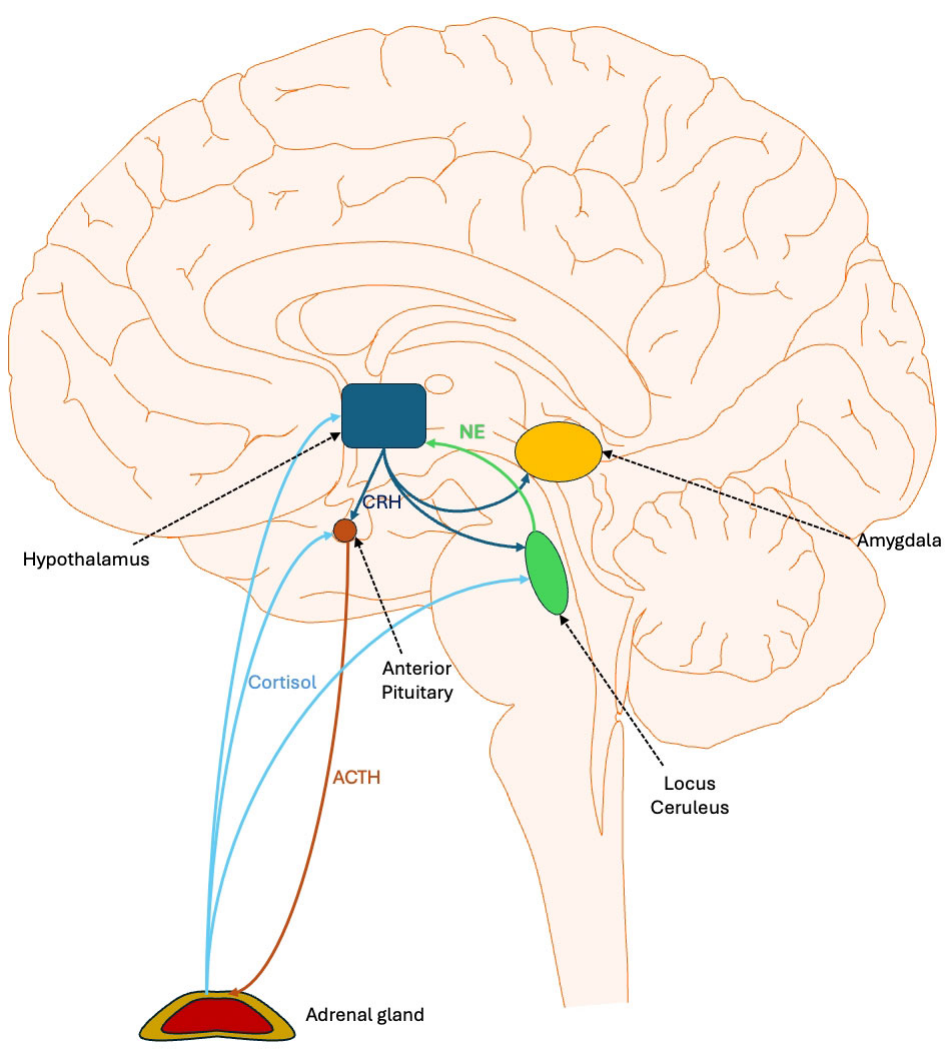
Central regulation of stress response: the hypothalamic pituitary adrenal axis and related structures. Stress activates the ventromedial nucleus of the hypothalamus to release corticotropin-releasing hormone (CRH). CRH travels through the hypophyseal portal system, a network of blood vessels connecting the hypothalamus and the anterior pituitary gland, to stimulate the release of adrenocorticotropic hormone (ACTH). This hormone then travels in the peripheral circulation to the adrenal cortex, where it triggers the release of cortisol. The latter binds to glucocorticoid receptors in various brain areas, thereby dampening the stress response. In particular, it inhibits the production of CRH and ACTH by neurons. CRH also projects to the locus coeruleus, which returns norepinephrine innervation to the hypothalamus, forming a positive feedback loop that is dampened by cortisol. CRH also innervates the amygdala, which mediates some of the emotional response to stress.

The impact of ACEs varies depending on factors such as the timing of exposure (e.g., early vs. later childhood), the nature of the adversity (e.g., abuse vs. neglect), and individual characteristics like sex (5, 19). Yet, trauma that occurs during sensitive periods of brain development tends to have more severe and enduring effects. Cumulative exposure to multiple adversities further compounds psychological and physiological burdens, leading to elevated risks for depression, suicidality, and other mental health conditions (20–22). ACEs can keep the brain’s stress-response system in a prolonged state of activation, which can lead to changes in gene expression (23). Rather than returning to baseline after a threat passes, children repeatedly exposed to trauma continue to secrete stress hormones, such as corticotropin-releasing hormone (CRH), leading to chronic hyperarousal (2, 24, 25) (**Figure 1**). This persistent stress disrupts the development of key brain regions involved in memory, attention, emotional regulation, and learning (2).

Chronic stress alters neurochemical pathways; for example, it increases dynorphin levels, which activate kappa opioid receptors (KORs), which inhibit dopamine release and contribute to depressive symptoms, particularly anhedonia (26–28). In addition to impairing brain development, prolonged early stress can trigger neuroinflammation, microglial activation, and epigenetic changes such as DNA methylation and altered microRNA (miRNA) expression (29). These biological mechanisms contribute not only to mental illness but also to substance abuse, high-risk behavior, chronic disease (9, 11, 18), and suicide.

In summary, chronic stress during early developmental stages leads to long-lasting disruptions in neurobiological systems that regulate emotion, cognition, and stress. These changes underlie an increased vulnerability to depression and other health outcomes through mechanisms involving hormonal imbalance, immune dysregulation, neuroinflammation, and structural brain alterations.

## Childhood trauma and the escalating mental health crisis in the United States

According to the CDC (30), between August 2021 and August 2023, 13.1% of individuals aged 12 and older in the U.S. experienced depressive symptoms during any given two-week period. This means that approximately 1 in 8 people met criteria for depression within that timeframe. Rates were especially high among females, younger individuals, and those living below the poverty line; 22.1% of those in poverty reported depressive symptoms, suggesting a strong link between economic hardship and mental health.

Over the past decade, depression has risen sharply, increasing from 8.2% in 2013–2014 to 13.1% in 2021–2023, a nearly 60% increase. Among those affected, 87.9% reported that their symptoms interfered with work, home, or social functioning. Despite this growing burden, more than 60% of individuals with depression did not receive treatment in the past year. These trends highlight an urgent need to expand access to mental health services and preventive care (30).

A growing body of research has identified ACEs as significant risk factors for both depression and suicidal behavior (31–34). ACEs are alarmingly common, with roughly one-third of the North American population reporting some form of childhood maltreatment (35, 36). ACEs have been associated with the earlier onset, severity, and chronicity of multiple psychiatric disorders, as well as higher rates of comorbid conditions and lower treatment response (37–39). Additionally, ACEs increase the risk of suicide attempts in adulthood by two to three times (40). Importantly, these effects may be mediated not only through the development of mental illness but also through intermediate disruptions in emotional development, such as attachment insecurity (32, 41, 42). ACEs are therefore considered an early risk factor, meaning its influence extends long after the trauma itself, unlike more immediate triggers (43, 44). The suicide model proposed by Turecki and Brent (43) underscores this, identifying childhood trauma as a persistent individual-level risk factor for suicide through mechanisms such as heightened emotional dysregulation and stress sensitivity.

Merrick et al. (45) conducted a multivariate analysis demonstrating that specific ACEs, including sexual abuse, physical abuse, household substance abuse, household mental illness, and emotional neglect, were all associated with greater risk of adult depression, suicide attempts, substance use, and alcohol misuse. Notably, sexual abuse emerged as the most impactful predictor, being significantly linked to all four adverse adult mental health outcomes (45). ACEs are strongly associated with long-term disruptions in emotional regulation, cognitive functioning, and neurodevelopment, significantly increasing the risk of psychiatric and physical health disorders across the lifespan (46–49). These findings emphasize the cumulative and long-lasting emotional toll of childhood trauma and highlight the need for early identification and trauma-informed care.

## Early-life adversity and dysregulation of the stress response system

Stress, particularly when experienced during early development, can induce lasting alterations in the amygdala, the brain region central to processing fear and emotional responses (50, 51). Stress activates the hypothalamic-pituitary-adrenal axis (HPA). The hypothalamus releases corticotropin releasing hormone (CRH), which travels to the pituitary that releases adrenocorticotrophic hormone (ACTH), which stimulates the adrenals to release cortisol (52, 53). The activation of the hypothalamus occurs primarily via norepinephrine (NE) originating in the locus ceruleus (LC) (54). While we commonly think of the actions of CRH being limited to the HPA, in fact, CRH receptors (e.g., CRHR1) are widely distributed across the brain, particularly in stress-responsive regions including the hypothalamus, hippocampus, amygdala, and locus ceruleus (55) (**Figure 1**). CRH projections originating in the hypothalamus innervate the LC and enhance NE release. This, then, forms a positive feedback loop with the hypothalamus (56). Early maternal separation, a model of early life stress in mice, permanently increases the density of CRHR1 in the amygdala, prefrontal cortex, and hippocampus (57). CRH makes the amygdala, particularly the central nucleus (CeA), more sensitive to emotional and threatening situations and it is involved in fear-related behavior, memory of aversive events, and fear conditioning (58).

Stressful experiences initially activate the limbic system, which signals the hypothalamus to release CRH and vasopressin (53, 59). Cortisol helps the body manage prolonged stress by regulating metabolism, immune function, and energy use (53). Simultaneously, the sympathetic nervous system is activated, which releases epinephrine and norepinephrine (NE) from the adrenal medulla (60). These hormones initiate the fight-or-flight response, increasing heart rate, alertness, and blood flow to muscles (60). This response also temporarily boosts brain function, particularly in the prefrontal cortex (PFC), which is involved in decision-making and emotion regulation (61, 62).

Even after the stressor resolves, the amygdala may continue to overreact to minor triggers, increasing the risk for anxiety, emotional dysregulation, and depression (23, 63, 2011). Animal studies confirm that early or chronic exposure to glucocorticoids leads to structural and functional changes in the amygdala that persist into adulthood (64–66). For example, research on children adopted after spending early childhood in orphanages found enlarged amygdala volumes, consistent with findings from animal models (67, 68). Interestingly, these studies did not report corresponding changes in the hippocampus (67, 68). Even after adoption into nurturing families, these children retained enlarged amygdala volumes (68, 69), suggesting that early adversity can produce enduring changes in amygdala structure that may not be fully reversed by later positive environments. Supporting this, animal studies have shown that stress-induced growth in the amygdala tends to persist, while other regions, such as the hippocampus, often exhibit recovery after the stress ends (68). One hypothesis is that this enduring amygdala enlargement reflects a biological adaptation to early danger, essentially keeping the brain on high alert in anticipation of future threats (68, 69). Over time, this heightened sensitivity can lower the threshold for fear responses, particularly when stress is frequent or occurs during critical developmental periods. Even after the stressor is removed, the amygdala may remain hyperreactive to minor triggers, increasing vulnerability to anxiety, emotional dysregulation, and other mental health conditions (68, 70). This persistent hyperreactivity may be partly explained by stress-induced impairments in GABAergic inhibition within the amygdala, especially a reduction in tonic inhibition, which usually helps suppress excessive neuronal firing and maintain emotional balance (71).

The amygdala is also part of a broader neural network that includes the ventromedial prefrontal cortex (vmPFC) and the hippocampus, regions that collaborate to regulate emotional responses and learning (72–75). The vmPFC exerts inhibitory control over the amygdala, while the hippocampus and amygdala co-regulate each other during emotionally salient experiences, allowing contextual memory to shape emotional responses (70, 76–78). However, stress – especially chronic or experienced early in life – can disrupt this finely tuned system (79). In adults, stress weakens the vmPFC’s capacity to regulate fear, thereby impairing processes such as fear extinction learning. This may occur through mechanisms such as decreased levels of brain-derived neurotrophic factor (BDNF), resulting in reduced synaptic plasticity and structural remodeling of neurons (80, 81). As a result, the amygdala may remain hyper-responsive to perceived threats, even in the absence of actual danger (82). Moreover, early life adversity appears to alter the structural and functional development of the vmPFC, suggesting that stress during sensitive developmental windows can lead to long-term disruptions in the connectivity and balance within the emotion-regulation network (83).

Dopamine, originating predominantly in the ventral tegmental area (VTA), is an important component of reward-related behavior, particularly incentive motivation, which is the drive to pursue reward, by altering the perceived valuation of a reward cue (84). Both acute and persistent stressors affect dopamine dynamics, but in opposing manners. An acute stress increases dopamine release, which helps to motivate the fight-or-flight response; that is, exerting energy to reduce or eliminate the stressor. By contrast, chronic stress dampens dopamine release by at least two mechanisms. Persisting stressors increase the release of striatal dynorphin, which activates presynaptic kappa receptors on striatal dopamine neurons (85). Stress also stimulates the amygdala to activate CRH projections to both the VTA and striatum, dampening dopamine release (86). ACEs are associated with altered striatal dopamine regulation, including an increase in striatal dopamine release (87), a blunted reward response to dopamine signals, and a blunted connectivity between VTA and striatum (88). This may underlie trait anhedonia, a key risk factor for depression (89).

ACEs similarly impact central serotonin (5-HT) function. For example, mice pups exposed to early life stressors exhibited an increase in anxiety-related behaviors and reduced functional connectivity of raphe 5-HT neurons (90). Prior trauma exposure, including early life trauma, is associated with altered 5-HT receptor expression, particularly 5-HT_1A_ receptors, which may alter subsequent risk of mood and trauma-related disorders (91). Alterations in 5-HT dynamics related to ACEs may contribute to heightened risk for mood, anxiety, and trauma disorders.

## Structural and functional brain changes following early-life and chronic stress: insights from human and animal studies

In parallel with amygdala alterations, early life adversity also affects other key brain regions involved in mood regulation, particularly the prefrontal cortex (PFC) and hippocampus (92, 93). Both individuals with major depressive disorder (MDD) and animal models of early or chronic stress exhibit reduced volume and neuronal atrophy in these areas (94–96). Functional imaging often shows decreased connectivity between the PFC, hippocampus, and associated regions (97), although increased connectivity in some areas suggests a more complex pattern of dysregulation. For example, adults with PTSD related to childhood maltreatment show heightened amygdala and hippocampal activation during emotional tasks, reflecting increased engagement of affective networks (98). Resting-state studies indicate that childhood abuse correlates with increased connectivity within the salience network (SN) and enhanced coupling between SN and the default mode network (DMN) (99). Additionally adverse childhood experiences are associated with stronger functional connectivity within the medial prefrontal cortex (mPFC) and between mPFC and other frontal regions, which may relate to repetitive self-referential processing, such as rumination (100). On the other hand, in animal models, in the Unpredictable Postnatal Stress (UPS) model, mice show reduced PFC volume and disrupted connectivity in the corpus callosum – findings that mirror structural abnormalities seen in trauma-exposed humans, including reduced corpus callosum volume and fractional anisotropy (FA) (19, 96).

Chronic stress induces dendritic atrophy and reduces spine density primarily in the apical dendrites of pyramidal neurons in layers II/III and V of the medial prefrontal cortex (mPFC), a brain region essential for decision-making, emotional regulation, and executive function (101–106). This structural remodeling impairs synaptic signaling by weakening excitatory postsynaptic potentials, partly due to the disruption of neurotransmitter systems, including serotonin (5-HT) and hypocretin (orexin), which are critical for maintaining synaptic strength (97, 107). The resulting reduction in synaptic connectivity and neurotransmitter sensitivity may contribute to the decreased size of the PFC and hippocampus observed in chronic stress. It may underlie the limited efficacy of selective serotonin reuptake inhibitors (SSRIs) in some patients, particularly those with a history of ACEs (97). Even short-term stress has measurable effects; just one week of restraint stress (20–30 minutes per day) can shrink pyramidal neurons in the PFC (61). Such rapid changes demonstrate how even brief stress exposures can compromise PFC integrity.

Postmortem studies provide further evidence of structural damage. Neurons often show signs of atrophy rather than a reduction in number in the dorsolateral PFC (dlPFC) and hippocampus of individuals with MDD (97, 108). Pyramidal neurons exhibit shrinkage, and GABAergic interneurons, which regulate excitatory signaling, are often reduced in number (97, 109). These findings point to a pattern of synaptic loss, neurotransmitter imbalance, and glial cell reduction underlying MDD. In humans, prolonged exposure to chronic stressors, whether social, environmental, or occupational, may lead to comparable atrophy, contributing to the impaired emotion regulation and decision-making commonly observed in depression (97).

Nonetheless, discrepancies between species and methodological variability highlight the need for cautious interpretation and more refined cross-species approaches. Longitudinal human studies, combined with advances in neuroimaging and molecular techniques, are essential for identifying sensitive developmental windows and potential mechanisms of resilience (110). By deepening our understanding of how stress shapes the brain, we can more effectively develop targeted interventions to prevent or mitigate psychiatric disorders stemming from early adversity.

## Chronic stress, synapse loss, and depression

Brain-derived neurotrophic factor (BDNF) is a crucial extracellular signaling protein belonging to the neurotrophin family, which supports neuronal development, survival, and plasticity (111). It is the most abundant neurotrophin in the central nervous system, highly expressed in key brain regions involved in mood regulation such as the hippocampus, amygdala, and prefrontal cortex (112, 113). BDNF primarily acts by binding to and activating the tropomyosin-related kinase B (TrkB) receptors, triggering pathways that promote neuronal growth, differentiation, and synaptic plasticity, promoting stress resilience (114, 115). These mechanisms are essential for neuronal survival, synaptic plasticity, maintenance, and growth. Under conditions of chronic stress, BDNF expression declines markedly in the PFC and hippocampus, leading to synaptic atrophy and behavioral symptoms of depression (116, 117). The effects of BDNF are region-specific: while reduced levels in the cortex and hippocampus are detrimental, increased BDNF in the mesolimbic dopamine system may worsen mood-related symptoms (116). The resulting synaptic impairment involves multiple interrelated mechanisms. One is the suppression of BDNF–TrkB signaling; under normal conditions, BDNF binds to TrkB receptors to activate pathways such as ERK and Akt, activating the mammalian target of rapamycin complex 1 (mTORC1), which drives synaptic protein synthesis, promoting dendritic spine and synapse formation, and maintaining synaptic structures (118). Chronic stress disrupts this signaling, compromising synaptic integrity (119, 120). Stress also dysregulates glutamate transmission by accelerating the degradation of glutamate receptors in the PFC, impairing excitatory neurotransmission and cognitive function (121). In addition, stress increases transcriptional repressors that suppress the expression of genes involved in synaptic maintenance and plasticity, further contributing to the loss of synapses (122). The molecular disruptions do not act in isolation. Instead, they are part of a broader cascade involving neuroendocrine imbalances and abnormalities in excitatory signaling. Chronic stress exerts profound effects on glutamatergic neurotransmission and adrenal steroid release, two closely linked systems that jointly shape synaptic plasticity and neuronal health.

## Glutamate, glucocorticoids, and the neurochemical cascade of chronic stress

In the early stage of acute stress, glutamate release in the prefrontal cortex (PFC) enhances focus, vigilance, and executive functioning (123). However, with prolonged or repeated exposure to stress, this same excitatory signaling becomes maladaptive (123, 124). Persistently elevated glutamate levels disrupt neural homeostasis, weaken synaptic integrity, and impair cognitive function (123, 124). Over time, sustained glutamate elevation becomes neurotoxic, resulting in dendritic atrophy, synaptic loss, and cognitive decline, hallmarks of depression-related neuropathology (97, 124).

Concurrently, the HPA axis becomes dysregulated, failing to shut off in over half of individuals with depression. This results in persistent high levels of stress hormones that contribute to further brain injury and depressive symptoms (97). Exposure to chronic or repeated stress leads to the release of adrenal steroids that affect the shape and structure of neurons, particularly in the hippocampus, a region essential for memory and emotion (97). Notably, studies show that blocking the n-methyl-D-aspartic acid (NMDA) receptors or stopping adrenal steroid production can prevent stress-related brain changes (125).

Normal neurotransmission also becomes disrupted as extracellular glutamate levels rise under chronic stress (126). To counteract potential excitotoxicity, Astrocytes play a major role in removing excess glutamate from the extracellular space through glutamate transporters that re-uptake the neurotransmitter, while glutamate receptors on cell surfaces detect and respond to extracellular glutamate (127). However, when glutamate accumulates outside the synapse, it activates extrasynaptic NMDA receptors, which suppress CREB signaling and initiate neurotoxic cascades, in contrast to synaptic NMDA receptors that promote survival and plasticity (128, 129), This receptor-location-dependent signaling shift contributes to dendritic atrophy and functional decline observed in stress-related disorders such as depression.

The CA3 region of the hippocampus offers particularly compelling evidence of how chronic stress alters glutamate transmission and neuronal structure (130). In the CA3 region of the hippocampus, glutamate release by mossy fibers – a key excitatory pathway – plays a pivotal role in stress-induced neuronal remodeling (131). This process is tightly regulated by adrenal steroids, such as cortisol, which modulate glutamate release (130). After 21 days of chronic restraint stress (CRS), animal studies show several markers of enhanced glutamatergic activity: (1) depletion of clear synaptic vesicles in mossy fiber terminals, suggesting excessive neurotransmitter release; (2) increased expression of presynaptic proteins involved in vesicle trafficking and release; and (3) greater mitochondrial density within terminals, indicating elevated energy demands to sustain neurotransmission (132). High levels of glucocorticoids synergize with excitatory amino acids, such as glutamate, to exacerbate excitotoxic damage —a process linked to impaired glucose uptake and reduced energy metabolism in neurons (133). This metabolic disruption limits the brain’s capacity to handle prolonged excitation, making neurons more vulnerable. Interestingly, this relationship follows an inverted U-shaped dose-response curve: at physiological levels, glucocorticoids translocate to mitochondria, where they reduce oxidative stress and support cellular resilience. However, during prolonged or excessive stress, this protective mechanism fails, leading to increased oxidative and excitotoxic damage (134). Mitochondria play a central role in both energy production and cellular stress responses, and their sensitivity to glucocorticoid signaling is crucial in determining whether the brain adapts successfully or deteriorates under chronic stress (135).

The glutamate transporter-1 (Glt-1), which clears excess glutamate from synapses, is upregulated in the hippocampus under stress (136). However, this regulation is biphasic: low to moderate glucocorticoid levels may enhance Glt-1 expression, but very high or prolonged exposure can suppress it (137). This highlights how timing and intensity of glucocorticoid exposure produce different neurological outcomes (137).

Interestingly, glucocorticoids can exert beneficial effects under certain conditions. For instance, they increase levels of cocaine amphetamine regulated transcript (CART) in the hippocampus during chronic stress – a change linked to reduced anxiety, possibly through modulation of hypothalamic stress circuits (138). This highlights the complex and context-dependent nature of glucocorticoid activity in the brain. In addition to synaptic regulation, glucocorticoids activate microglia, the brain’s resident immune cells, contributing to neuroinflammation in stress-sensitive regions such as the hippocampus, PFC, and amygdala (53).

Other molecules such as corticotropin-releasing factor (CRF), tissue plasminogen activator (tPA), and BDNF also play key roles in stress induced brain remodeling (139). CRF increases the expression of tPA in the amygdala, a change linked to anxiety-like behaviors and dendritic spine loss in both the amygdala and hippocampus. Notably, mice lacking tPA are protected from these stress-induced changes, suggesting that elevated tPA contributes to cognitive and synaptic impairments under chronic stress (140–143). Despite its harmful effects under excessive stress, tPA also plays a physiological role in neuronal plasticity. Specifically, it catalyzes the conversion of pro-BDNF to mature BDNF, which activates TrkB receptors and downstream pathways such as MAPK/Erk1/2 – promoting synaptic remodeling and modulating emotional behavior (144, 145).

## GSK3: A central player in synaptic pruning and depression

While tPA promotes the maturation of BDNF, chronic stress often leads to impaired BDNF signaling despite this compensatory mechanism. One key downstream target affected by reduced BDNF-Akt signaling is glycogen synthase kinase 3 (GSK3), an intracellular enzyme critical for synaptic plasticity and neuronal function (146–148). GSK3 plays a central role in synaptic deconsolidation or pruning by regulating the cycling of glutamate receptors, particularly the α-amino-3-hydroxy-5-methyl-4-isoxazolepropionic acid (AMPA) and NMDA receptors (149). Under normal physiological conditions, GSK3 is inhibited by Akt and activated by protein phosphatase 1 (PP1), maintaining synaptic stability (97). However, in chronic stress and depressive states, this inhibition is lost, leading to GSK3 overactivation, which contributes to dendritic spine loss in regions such as the prefrontal cortex and nucleus accumbens (150). Postmortem studies show elevated GSK3 activity in individuals with MDD and bipolar disorder, and animal models demonstrate that GSK3 inhibition in the nucleus accumbens produces antidepressant effects (150, 151). Lithium, a mood stabilizer widely used in bipolar disorder, is a potent inhibitor of GSK3 and has been shown to enhance the efficacy of some other antidepressants in MDD (152). These findings highlight GSK3’s role in depression-related neuroplastic changes and its promise as a therapeutic target. Interestingly, lithium has little if any antidepressant effects on its own, suggesting that just inhibiting GSK3 may not be sufficient for an antidepressant action. However, since antidepressants act via neurotrophic mechanisms, inhibition of the antineurotrophic effects of GSK3 may enhance antidepressant effects.

Beyond these synaptic and intracellular alterations, chronic stress also activates neuroimmune pathways. Microglia, the brain’s resident immune cells (153), play a pivotal role in mediating inflammation and structural remodeling, especially in stress-sensitive brain regions.

## Microglia in development and disorder: sensing stress and pruning synapses

As the brain’s resident immune cells, microglia are essential for healthy development and function (154). They contribute to neurogenesis, apoptosis, axonal growth, myelination, and immune responses to injury and infection (19, 155–158). A key function is synaptic pruning – the elimination of unnecessary or damaged synapses – which supports synaptic plasticity, refines neural circuits during development, and helps maintain cognitive function throughout life (159, 160). In addition to these structural roles, microglia secrete signaling molecules such as BDNF, transforming growth factor-beta (TGF-β), and complement proteins that regulate synaptic plasticity and behavior by shaping neural circuits (53, 158). However, this same sensitivity to external stimuli – such as sensory deprivation or early life stress – can render them vulnerable to dysfunction (19, 155).

Disruptions in microglial function may contribute to long-term cognitive and mental health challenges in individuals exposed to childhood adversity (19, 155). Early life stress has been shown to impair microglial pruning, resulting in excess synapses and abnormal brain connectivity – changes that likely underlie persistent cognitive and behavioral difficulties (19, 96, 161–163).

Chronic stress can drive excessive microglial activation, shifting these cells from a supportive to a pro-inflammatory, neurotoxic state. This dysregulated activation is linked to synaptic loss, reduced dendritic spine density, and impairments in memory and emotional regulation (164). Notably, interventions that restore IL-10 levels (an anti-inflammatory interleukin) or deplete microglia using PLX5622 reverse these memory and synaptic deficits, highlighting the critical role of microglia in depression-related cognitive impairment (164).

Microglia also contribute to the cognitive and emotional symptoms observed in PTSD. Both human patients and animal models show increased brain inflammation, including elevated TNF-α and IL-1β levels in the hippocampus. In a PTSD mouse model, microglia proliferate and adopt an activated morphology characterized by enlarged cell bodies, simplified branching, and dendritic spine loss in the hippocampal CA1 region – changes that likely underlie memory deficits and emotional dysregulation (165, 166). Importantly, these same studies suggest that chronic or dysregulated activation disrupts this balance – underscoring microglia’s dual role as both protectors and potential drivers of neuropathology.

## Mechanisms and functions of microglial synaptic pruning

Microglia contribute to synaptic plasticity by regulating key processes such as long-term potentiation (LTP) and long-term depression (LTD), which respectively strengthen or weaken synapses to encode learning and experience (167, 168). Through continuous monitoring of synaptic activity, microglia selectively preserve or eliminate synapses based on their functional relevance (158), thereby enabling dynamic synaptic remodeling that supports essential cognitive processes such as learning, memory, and behavioral adaptation (169, 170).

One major mechanism underlying microglia-mediated synaptic pruning is the classical complement cascade. In this pathway, proteins such as C1q, C3, and CR3 tag weak or redundant synapses for elimination, facilitating activity-dependent refinement of neural circuits (171–173). The C1q-dependent mechanism is especially important in memory-encoding engram neurons; its inhibition preserves synapses and enhances memory retrieval (158, 174). Blocking C1q signaling in the hippocampus also prevents synaptic degradation and reduces depressive-like behaviors in chronically stressed mice (175).

Complement component C3 is similarly pivotal in both developmental and stress-related pruning. Chronic stress increases C3 expression in the medial prefrontal cortex (mPFC), leading to excessive synaptic elimination by microglia. This disrupts connectivity between the mPFC and key mood regulating areas such as the anterior cingulate cortex, septum, and dorsal raphe nucleus, contributing to depression like behaviors (97). Notably, C3 knockout mice retain synaptic integrity and display resilience to stress-induced behavioral deficits – through complete C3 loss may also impair normal immune and synaptic functions (175–178). These findings highlight the pathological role of dysregulated complement signaling and suggest that targeted inhibition of C3 could offer therapeutic potential for stress-related mood disorders.

Beyond the classical complement cascade, other signaling pathways regulate microglial pruning. One such mechanism involves CD47 and SIRPα, which act as inhibitory signals that protect healthy synapses from inappropriate elimination by microglia (171–173). CD55, an inhibitor of this protective pathway, promotes pruning between engram cells in the dentate gyrus by allowing microglia to eliminate synapses that would otherwise be spared (158, 174). This leads to increased synaptic turnover and may influence memory retrieval by modifying engram reactivation (158, 174).

## Microglial synaptic pruning and stress-induced dysregulation

### TREM 2 – phosphatidylserine signaling and stress-induced impairment

Microglia eliminate dysfunctional synapses by recognizing phosphatidylserine (PS), a lipid exposed on compromised neuronal membranes, through the triggering receptor expressed on myeloid cells 2 (TREM2) (19, 158, 162, 179). The PS-TREM2 signaling pathway is crucial for synaptic refinement in brain regions such as the hippocampus and dorsal lateral geniculate nucleus, where pruning is essential for proper circuit maturation (158, 179). Notably, early-life stress disrupts this mechanism. Dayananda et al. (19) demonstrated that limited bedding (LB) and unpredictable postnatal stress (UPS) decrease surface TREM2 protein levels in microglia without affecting TREM2 mRNA expression, indicating post-transcriptional regulation. Consequently, impaired TREM2 signaling results in deficient synaptic pruning and an excess of dendritic spines on CA1 pyramidal neurons in the hippocampus, potentially leading to reduced neural efficiency and cognitive deficits later in life (19).

### Stress, microglial immune activation and neuroinflammation

In addition to synaptic pruning, microglia serve as the brain’s primary immune sentinels (154). They phagocytose cellular debris, respond to injury, and express immune markers such as MHC-II, CD80, and CD86, facilitating communication with peripheral immune cells (180). However, when excessively activated, microglia can drive a proinflammatory state that impairs neural function, highlighting their dual role as both protectors and potential contributors to neurodegeneration (53).

Stress activates the sympathetic nervous system, releasing norepinephrine, which binds to β2- adrenergic receptors on microglia. This activation triggers the production of inflammatory cytokines such as interleukin-1β (IL-1β) (181) and CCL2, which further recruit peripheral immune cells into the brain, amplifying neuroinflammation (53, 182). This inflammatory cascade disrupts synaptic function and plasticity. Notably, pharmacological blocking β-adrenergic receptors has been shown to reduce IL-1β expression and microglial activation, suggesting a promising strategy for mitigating stress-induced neuroinflammation (53, 182).

### Molecular regulation of microglial synaptic pruning

Microglial synaptic pruning and phagocytosis are partly regulated by the CX3CR1/CX3CL1 signaling pathway, in which the microglial receptor CX3CR1 binds its neuronal ligand CX3CL1 (fractalkine) to enable direct neuron–microglia communication (158, 183). This signaling axis plays a critical role in guiding microglia to eliminate synapses selectively during brain development and in response to environmental changes. Disruptions in this pathway impair synaptic refinement and may contribute to abnormal neural connectivity in both neurodevelopmental and stress-related disorders (158).

In addition to synaptic pruning, CX3CR1/CX3CL1 signaling shapes microglial interactions with oligodendrocyte progenitor cells (OPCs) (184). In CX3CR1 knockout mice, microglia retain contact with OPCs but exhibit reduced phagocytic activity, suggesting that CX3CR1 is crucial for engulfment rather than target detection alone (158, 185–187). Deleting either CX3CR1 or its ligand CX3CL1 also impairs synapse elimination following sensory deprivation, such as whisker cauterization in rodents (188), further underscoring the importance of this pathway in activity dependent neural remodeling (186, 189).

Early-life stress models, such as unpredictable postnatal stress (UPS) and limited bedding (LB), differentially influence microglial function through distinct molecular mechanisms. In the UPS model, microglia exhibit increased expression of pruning-related genes – including C1q, MERTK, and SIRPα – which correlates with enhanced synaptic pruning, increased microglial volume, and greater synaptic engulfment (19). In contrast, the LB model is associated with elevated TNF and reduced NGF, which may disrupt synaptic development and impair cognitive outcomes.

Although both models increase CX3CR1 expression, possibly as a compensatory mechanism, this response appears insufficient: both UPS and LB ultimately lead to increased spine density. Notably, the UPS model also reduces IL-1α and IL-1β levels, further impairing neurogenesis and synaptic signaling. These distinct molecular profiles may help explain the divergent effects of early-life stress on hippocampal development and function (19).

To prevent excessive or inappropriate pruning, microglial phagocytic activity is tightly regulated by inhibitory signals, including CD47/SIRPα and CD200/CD200R, which protect functional synapses during development (173, 190–192). CD47, a protein expressed on neurons, binds to SIRPα receptor on microglia, suppressing phagocytosis and protecting synapses from inappropriate elimination.

Similarly, the neuronal glycoprotein CD200 binds to the CD200R receptor on microglia to suppress their phagocytic activity (192, 193). In CD200 knockout mice, microglia exhibit increased synaptic engulfment, particularly in response to amyloid beta, highlighting the loss of inhibitory control in the absence of this signaling pathway (192, 193).

Another important regulator is high mobility group box 1 (HMGB1), a nuclear protein released during stress or injury. Acting as a damage-associated molecular pattern (DAMP), HMGB1 triggers inflammation and microglial activation, making it a potential therapeutic target in neuroinflammatory and stress-related conditions (194, 195).

## MicroRNA as potential regulators of long-lasting neuroinflammatory and structural brain changes

MicroRNAs (miRNAs) are small, single-stranded RNA molecules that regulate gene expression post-transcriptionally by binding to messenger RNAs (mRNAs), thereby blocking their translation or promoting their degradation (196). Importantly, miRNA activity is also modulated by other non-coding RNAs, particularly long non-coding RNAs (lncRNAs), which can sequester miRNAs and prevent them from interacting with target mRNAs – thus indirectly preserving gene expression (197–199). Another class of small RNAs, small interfering RNAs (siRNAs), similarly mediate gene silencing through RNA interference (RNAi), although they differ from miRNAs in terms of biogenesis and target specificity (196, 200, 201).

miRNAs play essential roles in the central nervous system, contributing to neurogenesis, axonal guidance, synaptic development, and neuronal plasticity. Through these functions, they shape neuronal growth, connectivity, and long-term adaptability (198, 202–205).

To carry out these regulatory roles, miRNAs undergo a multistep biogenesis process. They are transcribed from miRNA genes in the nucleus by RNA polymerase II into primary transcripts (pri-miRNAs), which form characteristic hairpin loop structures (196). These pri-miRNAs are cleaved by the DROSHA-DGCR8 complex into precursor miRNAs (pre-miRNAs), which are then exported to the cytoplasm via Exportin-5 (206, 207).

Once in the cytoplasm, the pre-miRNA is further processed by the enzyme Dicer and its cofactor TRBP into a short RNA duplex. In some instances, Argonaute 2 (AGO2) may perform additional cleavage (206, 208, 209). The mature strand of this duplex is then incorporated into the RNA-induced silencing complex (RISC), while the passenger strand is degraded. Guided by the mature miRNA, the RISC complex binds to complementary target messenger RNAs (mRNAs), leading to translational repression and mRNA degradation (206, 208, 209).

### MicroRNA regulation of synaptic plasticity in major depressive disorder

MicroRNAs (miRNAs) are critical post-transcriptional regulators of synaptic plasticity. Disruption of key processing enzymes such as Dicer and DGCR8 impairs synaptic remodeling in mouse models, leading to structural abnormalities – such as reduced dendritic branching and elongated dendritic spines – accompanied by cognitive and behavioral deficits (198, 210).

Specific miRNAs, including miR-124, miR-132, miR-134, and miR-138, fine-tune the expression of plasticity-related genes such as ARC, CaMKIIα, LimK1, FMRP, CREB, and BDNF (198, 211). For instance, miR-134 negatively regulates LimK1, disrupting dendritic spine structure and weakening excitatory synaptic transmission (198, 212). Elevated miR-134 expression – observed in the hippocampal CA1 region of Sirt1-deficient mice – reduces CREB and BDNF levels, highlighting the role of miRNAs in neurotrophin-mediated synaptic regulation (198, 213).

Other miRNAs such as miR-9, miR-125a/b, and miR-188 epigenetically influence genes including REST, FXR1P, CAMKK2-AMPK, PSD-95, BCL-W, SYN-2, NRP-2, 2-AG, and BACE1, demonstrating the broad regulatory role of miRNAs in maintaining neuronal plasticity and cognitive function (198, 212).

More than 25 studies have reported altered miRNA expression in key brain areas affected by MDD, including the anterior cingulate cortex and Brodmann Areas (BA) 9, 10, 44, and 46, as well as the locus coeruleus (198). For example, Smalheiser et al. (214) identified 21 miRNAs significantly downregulated in the prefrontal cortex of individuals with MDD who died by suicide, many of which target genes critical for synaptic plasticity.

Extending these postmortem findings, later studies reported significant reductions in synaptosomal miRNAs – especially miR-508-3p and miR-152-3p – with miR-508-3p levels particularly diminished in suicide cases (198). These results support the hypothesis that suicidal depression is associated with widespread miRNA downregulation in brain circuits governing mood and cognition (214, 215).

Similarly, Lopez et al. (216) reported decreased expression of miR-1202 in Brodmann are (BA) 44 of individuals with MDD. Additional evidence implicates miR-124-3p, which was significantly reduced in BA 46 of MDD patients (217). Interestingly, fluoxetine treatment further reduced miR-124-3p expression, suggesting a potential role in antidepressant mechanisms (198).

In a study of the locus coeruleus in MDD patients who died by suicide, Roy et al. (217) identified 10 upregulated and 3 downregulated miRNAs. Predicted targets of these upregulated miRNAs included RELN, GSK-3β, MAOA, CHRM1, PLCB1, and GRIK1 – genes previously implicated in psychiatric disorders (198). Notably, several of these targets, particularly RELN, GSK-3β, and MAOA, showed reduced expression, supporting their potential involvement in the molecular mechanisms underlying depression and suicide (198).

Using microarray analysis, researchers identified several genes whose expression was inversely correlated with miR-1202, including metabotropic glutamate receptor 4 (GRM4), a gene implicated in antidepressant response (216). These results suggest that miR- 1202 may serve as a predictive biomarker for treatment outcomes in MDD.

Azevedo et al. (218) further investigated miRNA expression and found that miR-34a and miR-184 were significantly downregulated in individuals with MDD. They focused on miR-34a, which regulates genes such as NCOA1, NCOR2, and PDE4B – key players in glucocorticoid receptor signaling and synaptic cAMP regulation. *In vitro* studies confirmed that miR-34a overexpression reduced NCOA1 and PDE4B levels, supporting its role in disrupting stress hormone signaling and synaptic function, thereby contributing to the pathophysiology of MDD (198).

### miRNAs as biomarkers in depression, treatment response, and suicide risk

Many miRNAs are closely involved in regulating synaptic plasticity, neuroinflammation, and glucocorticoid signaling, all of which are disrupted in depression (219). Because of their specificity and regulatory power, these small non-coding RNAs are gaining attention not only as mechanistic contributors but also as potential biomarkers of psychiatric illness, particularly, MDD, bipolar, and schizophrenia (SZ) (220). Among the most studied are miR-124, miR-125, miR-29, miR-16, and miR-200, which are consistently associated with stress-related psychiatric conditions (206).

A notable study by Cattane et al. (221) found elevated levels of miR-125b-1-3p in both healthy individuals with a history of early-life stress and in patients with SZ. These results, validated in animal models and *in vitro*, suggest that early changes in miR-125b may lead to lasting disruptions in neuronal signal transduction via NMDA receptor subunit targeting – contributing to altered brain plasticity and psychiatric vulnerability (198, 222).

More recently, miRNA profiling has been used to differentiate between psychiatric conditions and predict treatment response. For example, Maffioletti et al. (223) analyzed blood samples from patients with MDD, bipolar disorder (BD), and healthy controls, identifying distinct expression patterns across groups. miR-24-3p, miR-425-3p, and members of the let-7 family were significantly altered in MDD, while miR-30e, miR-21– 3p, and miR-140–3p were associated with BD. KEGG pathway analysis identified Wnt and mTOR signaling as key biological targets of these dysregulated miRNAs.

Building on this, Lopez et al. (216) examined miRNA expression following duloxetine treatment using next-generation sequencing. They found that miRNAs involved in Wnt and MAPK signaling – including miR-146b-5p, miR-24-3p, and miR-425-3p – were downregulated post treatment but elevated in postmortem brains of suicide victims, suggesting these miRNAs may be linked to treatment resistance or elevated suicide risk.

### miRNAs in treatment response and peripheral biomarker potential

Further evidence from Roy et al. (224) and Lopez et al. (216) supports the role of several miRNAs–miR-146b-5p, miR-24-3p, and miR-425-3p-as potential treatment-response markers. These miRNAs decrease with escitalopram treatment but are elevated in postmortem brain samples of individuals who died by suicide. Functionally, they are linked to Wnt and MAPK pathways, which regulate synaptic plasticity, stress response, and mood. Consistent with this, rats with depression-like behavior showed reduced expression of Wnt pathway genes targeted by miR-128-3p, further emphasizing the significance of miRNA-pathway interactions in depression biology (224).

Importantly, some miRNA changes in the central nervous system are mirrored in peripheral blood, especially through exosomes (174). For example, miR-330-3p is upregulated in both the locus coeruleus and blood samples of MDD patients (198). Similarly, miR-124-3p and miR-19a-3p display comparable expression in brain regions and blood (217, 225) suggesting their potential as accessible biomarkers that reflect CNS pathology.

miR-124, one of the most abundant brain microRNAs, arises from three distinct genes encoding the same mature sequence and is essential for neurogenesis, neuronal differentiation, and synaptic plasticity (226–228).

Evidence suggests a context-dependent function in stress-related behaviors. In the hippocampus, miR-124 enhances resilience to chronic stress through posttranscriptional regulation of HDAC4/5 and GSK3β expression (229). In contrast, other studies show that miR-124a overexpression promotes depression-like behaviors, whereas its inhibition yields antidepressant-like effects, potentially via CREB1 and BDNF upregulation (230, 231).

Likewise, miR-19a-3p has been implicated in neuroinflammation through its interaction with tumor necrosis factor-alpha (TNF- α) (225, 232). In the dorsolateral prefrontal cortex (dlPFC) of individuals who died by suicide, both TNF-α and miR-19a-3p were elevated. Notably, similar increases were observed in the blood of depressed individuals with suicidal ideation, suggesting that miR-19a-3p may reflect central immune activation in peripheral samples (232).

Although miRNAs usually suppress gene expression, Wang et al. (225) showed a nonlinear interaction: the RNA-binding protein HuR binds to the 3′ untranslated region (3′UTR) of TNF-α mRNA, shielding it from miR-19a-3p-mediated repression. This may explain persistently high TNF-α levels despite elevated miR-19a-3p, emphasizing the context-dependent complexity of miRNA regulation.

Despite these regulatory intricacies, circulating miRNAs also hold promise as accessible biomarkers. A serum-based case-control study demonstrated that a panel of miR-16, miR-135a, and miR-1202 could distinguish individuals with MDD from healthy controls (233). The combined panel outperformed individual miRNAs in diagnostic accuracy.

This potential is reinforced by the role of extracellular vesicles (EVs) **–** membranous carriers of nucleic acids and proteins involved in intercellular communication (234). Because EVs can cross the blood brain barrier and reflect CNS pathology, they represent a promising platform for non-invasive diagnosis of neuropsychiatric conditions (235, 236).

However, a significant challenge lies in ensuring EV purity. EVs are inherently heterogeneous, and contamination or inconsistent isolation can compromise the specificity and reproducibility of miRNA profiling (237). To realize the full diagnostic utility of EV-based miRNA biomarkers, rigorous standardization in EV isolation and characterization is imperative.

Despite over a decade of research, the clinical application of miRNAs as diagnostic or prognostic tools remains in its early stages (198, 238). Genetic markers such as the 5-HT2A receptor and SKA2 gene have also been explored in relation to suicidal behavior (239, 240). While numerous biomarkers have been investigated, only a few – such as prolactin and thyroid hormone levels – have demonstrated consistent reliability in predicting behavioral outcomes like suicide attempts (241).

## DNA methylation and suicide risk in MDD

Building on the critical role of miRNAs as biomarkers for diagnosis and treatment, DNA methylation has also emerged as a key epigenetic mechanism implicated in the pathophysiology of trauma-related MDD and suicidal behavior (242, 243). Like miRNAs, DNA methylation modulates gene expression without altering the genetic code and may offer novel biomarkers for early detection and personalized treatment (244, 245).

DNA methylation refers to the addition of methyl groups to cytosine bases, particularly at CpG sites, leading to transcriptional repression when occurring near promoter regions (246). Abnormal methylation patterns have been increasingly associated with psychiatric conditions, especially MDD and suicidality (244, 247).

Early-life stressors, such as childhood abuse or neglect, can induce long-lasting epigenetic modifications, leaving molecular “scars” that increase lifelong vulnerability to depression and suicidal tendencies (64, 248). These epigenetic changes are particularly evident in brain regions involved in emotion regulation and stress processing, including the prefrontal cortex, hippocampus, and amygdala (249).

Specific methylation changes have been found in genes critical to neurotransmission and neuroplasticity, including *SLC6A4* (serotonin transporter), *BDNF* (brain-derived neurotrophic factor), and GABA A receptor subunits (245, 249, 250). For example, hypermethylation of the *SLC6A4* promoter is linked to reduced serotonin reuptake, while decreased *BDNF* expression through promoter hypermethylation is associated with impaired synaptic function (249). Hypermethylation of *TrkB1* and *BDNF* in suicide victims’ prefrontal cortices further supports this association (249). In addition, genome-wide studies have identified broader methylation alterations across hundreds of promoters involved in neurodevelopment, immune function, and synaptic signaling (251, 252). These epigenetic modifications predominantly localize near gene promoters and regulatory elements, including transcription start sites (TSS), 5′ untranslated regions (5′UTR), and 3′UTRs.

DNA methylation was first recognized in CpG-rich regions known as CpG islands – segments of DNA approximately 1,000 base pairs in length with a high frequency of cytosine-guanine dinucleotides (253–255). Roughly 70% of gene promoters are located within these islands, making them critical regions for transcriptional regulation. Because promoter methylation typically leads to transcriptional repression, CpG islands in promoters of actively transcribed genes are usually unmethylated to allow gene expression (253). Elevated methylation in these regions has been consistently observed in individuals with MDD and heightened suicide risk, implicating the silencing of genes critical for stress adaptation and emotional regulation (242, 245).

To further investigate these mechanisms, researchers performed protein–protein interaction (PPI) analyses on the top 100 differentially methylated CpG sites, identifying *CDH5*, *ACTN1*, and *GNA12* as hub genes in individuals with MDD, regardless of suicide history (245, 256, 257). These hub genes regulate key downstream targets including *RAPTOR*, *ADAMTS17*, and *HSP90AA1*. Specifically, *RAPTOR* is involved in excitatory neuronal activity and synaptic remodeling; *ADAMTS17* contributes to cognitive function through synaptic plasticity; and *HSP90AA1* modulates the HPA axis (178, 258–261). Notably, both *RAPTOR* and *ADAMTS17* are integral components of the mTOR signaling pathway, which interacts with AMPA receptors to support stress resilience (262, 263). Epigenetic silencing of these genes may impair neuroplasticity and reduce the ability to adapt to chronic stress, thereby increasing suicide risk among trauma-exposed individuals (264, 265).

Chromosome-specific methylation patterns have also emerged as significant in MDD and suicide. Alterations in methylation are particularly prominent on chromosomes 2, 3, and 4 (245). Chromosome 2 is primarily associated with increased gene silencing, whereas chromosome 3 shows more gene activation, offering insights into chromosome-level vulnerability to environmental and epigenetic influences (245, 266, 267). Some studies have reported reduced or unchanged methylation levels, likely reflecting differences in CpG site selection, analytic methods, and sample characteristics such as ancestry and tissue type (268).

### Trauma-associated methylation of glucocorticoid signaling genes

In addition to genes involved in synaptic plasticity, trauma-related methylation patterns frequently affect key components of the stress response system, particularly *NR3C1* and *FKBP5*, which modulate glucocorticoid signaling (269, 270). Increased methylation of *NR3C1*, which encodes the glucocorticoid receptor, has been consistently observed in children exposed to early-life adversity, suggesting disrupted stress feedback mechanisms and heightened emotional vulnerability (269, 271). In adults, findings have been more variable: some studies have reported increased *NR3C1* methylation following early-life adversity, while others have found hypomethylation associated with suicide or post-traumatic stress disorder (PTSD) (251, 272, 273).

IL-6, a cytokine induced by stress, is another molecule closely linked to the epigenetic mechanisms underlying depression. IL-6 promoter methylation in DNA extracted from buccal swabs was found to be lower in older adults (≥65 years) with major depressive disorder (MDD) compared to healthy controls (274). Notably, antidepressant use was independently associated with higher IL-6 methylation, supporting the potential role of IL-6 as a biomarker for both MDD and antidepressant response.

FK506 binding protein 5 (*FKBP5*) is a chaperone protein that protects the glucocorticoid receptor in the cytosol and modulates its sensitivity (275). Klengel et al. (276) were the first to report that childhood maltreatment is linked to decreased *FKBP5* methylation in individuals carrying the T risk allele. This gene–environment interaction was replicated in a follow-up study (277). However, other research has found no significant effect of maltreatment or genotype, and one study in young adults observed that childhood stressful events were associated with increased *FKBP5* methylation, which in turn mediated alterations in prefrontal brain activity (278). These mixed findings highlight the complexity of trauma-related epigenetic responses and reinforce the potential of *NR3C1* and *FKBP5* methylation as biomarkers of sustained stress exposure and elevated suicide risk.

Beyond specifying which genes or gene regions are hyper- or hypomethylated, several important considerations must be addressed. First, prenatal exposures can exert epigenetic effects on stress-related biological systems, potentially confounding associations observed in childhood (279). For example, intimate partner violence during pregnancy has been linked to increased *NR3C1* methylation in offspring during late childhood and adolescence (280). Similarly, maternal smoking and depression during pregnancy have been associated with altered methylation of placental stress-regulatory genes, including *NR3C1* and *HSD11B2*, the latter encoding an enzyme that inactivates cortisol (281–284). These findings suggest that associations between childhood maltreatment and DNA methylation may, in part, reflect prenatal environmental influences.

Second, sex differences may moderate the effects of trauma on DNA methylation. Future research should carefully consider the role of the sex of a child to avoid overlooking critical gene-by-sex interaction effects (268).

Finally, variation in tissue types used for methylation analysis also warrants attention. While blood, saliva, and buccal cells have all been utilized, most pediatric studies rely on saliva or buccal samples, with fewer examining methylation patterns in blood (285). This methodological heterogeneity may affect the consistency and comparability of results across studies.

Paoli et al. (286) reviewed and analyzed published studies investigating DNA methylation in depression, emphasizing its potential for future biomarker development and pharmacological intervention. Their meta-analysis identified four genes – BDNF, SLC6A4, FKBP5, and NR3C1 – with the highest consistency of methylation changes. Specifically, most studies reported increased methylation at BDNF, SLC6A4, and NR3C1, while FKBP5 typically showed decreased methylation associated with depression. DNA methylation thus represents a promising avenue for future research and clinical application. However, like miRNAs, its diagnostic and predictive utility remains under investigation. Continued research is essential, but current findings provide considerable hope for advancing precision medicine in psychiatry and stress-related disorders.

## Synaptic dysregulation in depression and suicide: the role of stress, epigenetics, and rapid-acting antidepressants

According to recent CDC data (287), depression affects 19.2% of adolescents aged 12 to 19, compared to just 8.7% of adults over 60. Certain groups, like adults with disabilities, are disproportionately affected – 28.2% take medication for depression, nearly three times higher than those without disabilities. In 2023, over 49,000 Americans died by suicide – one every 11 minutes (288). These alarming statistics make clear the urgent need for improved early detection and intervention strategies. Suicide risk is strongly associated with depression, substance use disorders – particularly alcohol use – and other comorbid psychiatric conditions (289).

Mounting evidence highlights ACEs as foundational contributors to long-term psychiatric vulnerability (290, 291). The effects of ACEs appear cumulative, with higher exposure linked to an increased risk of depression, anxiety, self-harm, and suicidality in adulthood (292–294).

In psychiatric clinical practice, assessing a patient’s suicidal ideation is ideal, but not always possible. According to Obegi (295), nearly 50% of individuals with suicidal ideation withheld this information from their healthcare providers. Given this barrier, systematically screening for psychiatric risk based on ACE history may help identify individuals at elevated biological and psychological risk – without relying on verbal disclosure. This emphasizes the need for objective, biologically based tools to support suicide prevention efforts (296).

MDD is increasingly understood as a condition rooted in disrupted synaptic function (108, 122, 297). Neuroimaging studies consistently demonstrate reduced volume in the prefrontal cortex and hippocampus – regions essential for emotion regulation, memory, and cognition – suggesting stress-related neuronal atrophy, particularly in cases of prolonged illness or delayed treatment (92, 93). Functional imaging further reveals altered connectivity between these regions and broader disruptions across neural circuits, potentially reflecting dysregulated reciprocal signaling (94, 95). These anatomical and functional abnormalities are now believed to arise from a cascade of molecular and cellular alterations induced by chronic stress. This cascade begins with glutamatergic overactivation and is compounded by glucocorticoid dysregulation, resulting in widespread disturbances to neuronal architecture and synaptic signaling (298, 299). In addition to impairing mitochondrial integrity and immune function, these changes promote region-specific remodeling of brain structures (300, 301). A key driver of this pathology is neuroinflammation: stress activates microglia and increases pro-inflammatory cytokine levels, leading to synaptic dysfunction and reduced neurogenesis (53). Together, these molecular disruptions converge to produce the brain atrophy and weakened synaptic connectivity consistently observed in MDD.

## Treatment

Early life trauma tends to reduce the responsiveness to standard antidepressant therapies (302). ACEs appear to induce anti-neurotrophic effects that are sufficient to counteract the pro-neurotrophic effects of regular oral antidepressants like the serotonin selective reuptake inhibitors. Over the last two decades, efforts have been made to develop a new generation of antidepressants with enhanced neurotrophic effects. The first of these, has been shown to improve the treatment of many patients with treatment-resistant major depression, including those with histories of trauma (303). Some more recent research suggests that ketamine may also be effective for PTSD (304).

**Figure 2:**
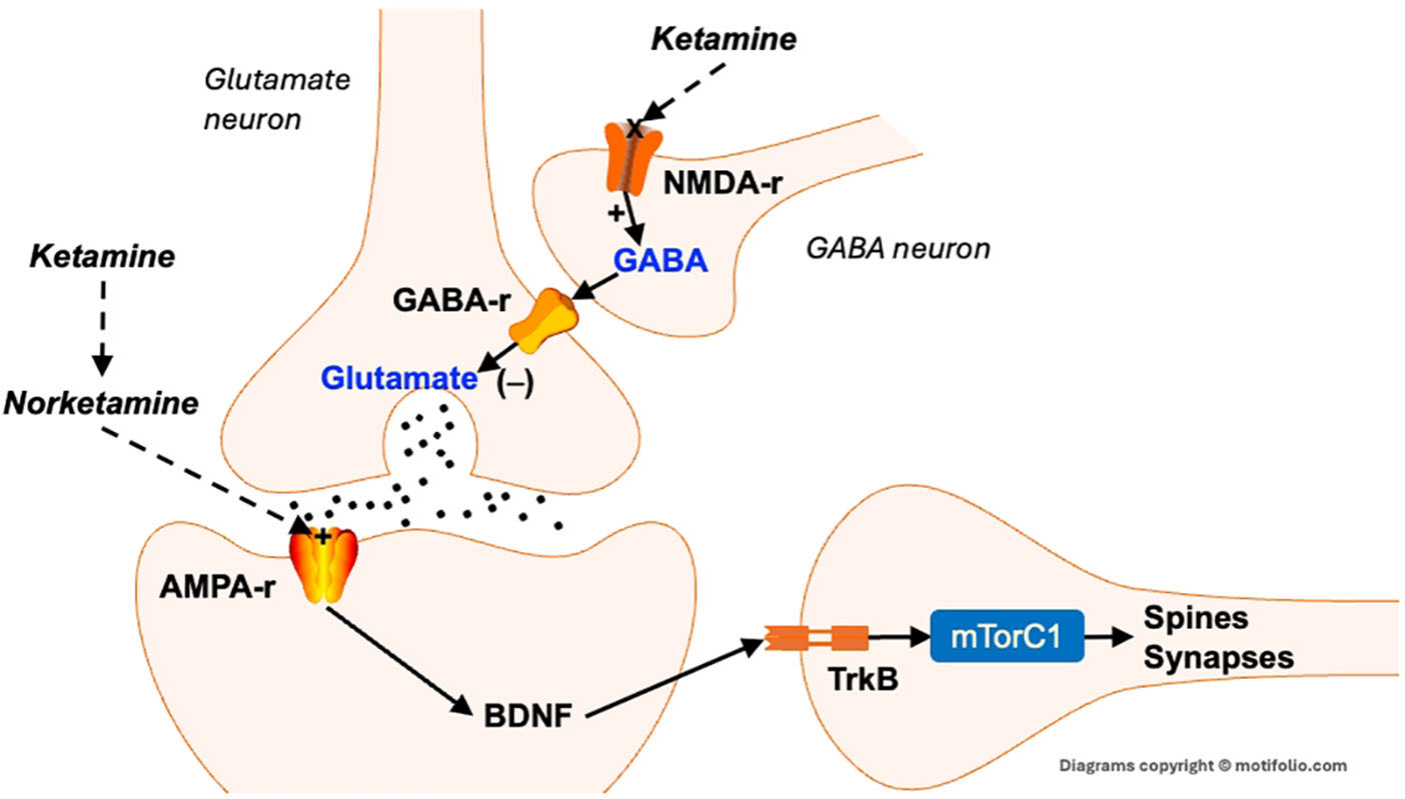
Neurotrophic effects of ketamine. Excess glutamate activates NMDA receptors on GABA-containing interneurons. This stimulates the release of GABA, which binds to GABA_A_ receptors that inhibit the release of glutamate. The blockade of NMDA receptors reduces GABA’s inhibition of glutamate neurons, leading to a rapid release of glutamate. This, in turn, activates post-synaptic AMPA receptors that, among other actions, cause the rapid release of BDNF. BDNF then binds to its receptor trk-B, which activates mTORC1, resulting in the rapid synthesis of spine and synaptic proteins. This then results in the formation (or maintenance) of dendritic spines and synapses. AMPA-r, α-amino-3-hydroxy-5-methyl-4-isoxazolepropionic acid receptor. BDNF, Brain-derived neurotrophic factor; GABA-r, Gamma amino butyric acid receptor; NMDA-r, N-methyl-D-aspartic acid receptor; TrkB, tropomyosin receptor kinase B.

In recent years, ketamine, an FDA-approved anesthetic and pain management agent, has emerged as a rapid-acting antidepressant by targeting the mTORC1 signaling pathway and downstream plasticity pathways (97). Ketamine rapidly induces robust antidepressant effects by blocking NMDA receptors, which triggers a surge in glutamate release and subsequent activation of AMPA receptors (305) (**Figure 2**). One of ketamine’s metabolites, N-desmethylketamine (norketamine), also directly activates AMPA receptors (306). AMPA receptor activation promotes the rapid release of BDNF, which then binds to its receptor, tropomyosin receptor kinase B (TrkB), initiating downstream signaling through the mTORC1 pathway that leads to spine and synapse formation (307). As a noncompetitive NMDA receptor antagonist, ketamine can produce antidepressant effects within hours, particularly in patients resistant to traditional treatments (303, 308).

In addition to promoting BDNF release, ketamine induces the expression of activity-regulated cytoskeleton-associated protein (Arc), inhibits glycogen synthase kinase-3 (GSK3), and reverses stress-induced synaptic loss in the medial prefrontal cortex (PFC) and hippocampus (149, 150, 309). These effects are dependent on intact BDNF and GSK3 signaling pathways – blocking either pathway eliminates ketamine’s behavioral and synaptic benefits in animal models (310–313). The resulting synaptogenesis enhances emotional regulation and is considered a core mechanism behind ketamine’s rapid antidepressant effects. However, despite its efficacy, ketamine’s clinical use is constrained by short-lived benefits, the need for repeated dosing, and side effects such as dissociation and psychosis (314). To address these challenges, several novel therapeutic agents are being developed to replicate ketamine’s rapid antidepressant properties while minimizing its adverse effects. These include AMPA receptor positive allosteric modulators (AMPA-PAMs), which enhance glutamatergic transmission and synaptic plasticity (315, 316) and NMDA subunit modulators.

The NMDA receptor is composed of four protein subunits, two NR1 and two NR2 (which can be further classified as NR2A, B, C, and D). There are also NR3 subunits that can be assembled into a complete NMDA receptor. NMDA receptor modulators such as apimostinel and zelquistinel also produce rapid antidepressant effects in animal models and early-stage human studies, but with improved safety profiles. Unlike ketamine – which directly blocks the NMDA receptor ion channel – these compounds selectively inhibit the NR2B subunit, a mechanism that may account for their reduced risk of dissociative side effects (317). Additional agents targeting the NMDA receptor or downstream effectors such as mTORC1 are also under development (315, 316). These next-generation, rapid-acting antidepressants represent a paradigm shift – moving beyond traditional monoaminergic strategies to directly enhance synaptic resilience.

## Conclusions

Collectively, the evidence presented in this paper demonstrates how adverse early experiences interact with neurobiological processes to increase susceptibility to depression and suicidality. A thorough understanding of these mechanisms is critical for designing more effective, biologically informed interventions. Looking ahead, integrating pharmacological advances with emerging epigenetic tools – such as microRNA profiling and DNA methylation analysis – holds promise for identifying at-risk individuals even before clinical symptoms manifest. These personalized strategies could transform psychiatric care from reactive crisis management into proactive prevention.

There are several limitations to this review. Although this review is broad in scope, it does not cover every possible mechanism. This was not a systematic review. We acknowledge that, even within the specific topics covered in this paper, there may be relevant information that was not addressed. We also did not cover the important area of biomarkers, given the length and complexity of the issues covered.

This review highlights the fact that emotional traumas experienced early in life have far-reaching effects that may last a lifetime. An integrative view is that many possible mechanisms, including DNA methylation, altered expression of both protein-coding genes and short regulatory RNAs like miRNAs, enhancement of pro-apoptotic mechanisms, synaptic remodeling, and others, have anti-neurotrophic effects that predispose to mood, anxiety, and trauma-related disorders and suicide across the lifespan. Novel treatments like ketamine must have profound pro-neurotrophic effects that supersede the blocks introduced by traumas. While ketamine is a very effective treatment, there are distinct disadvantages to ketamine, including the intravenous mode of delivery and significant side effects. Several NMDA-selective antagonists, including NR2B-selective NAMs, have failed in clinical trials for various reasons. A dextromethorphan and bupropion combination drug, which ostensibly targets NMDA receptors, has been approved for treatment-resistant major depression. Still, it is unclear if it is as effective as ketamine, particularly in people with a history of trauma. Time will tell if newer compounds like the “stinels” (apomostinel and zelquistinel) or onfasprodil that target the NMDA receptor, or mefluleucine (NV-5138) that completely bypasses upstream signal transduction mechanisms by directly activating mTORC1, will eventually make it through clinical trials for depression or other conditions.
